# Neuronal toll-like receptor 4 signaling induces brain endothelial activation and neutrophil transmigration *in vitro*

**DOI:** 10.1186/1742-2094-9-230

**Published:** 2012-10-03

**Authors:** Sophie Leow-Dyke, Charlotte Allen, Adam Denes, Olov Nilsson, Samaneh Maysami, Andrew G Bowie, Nancy J Rothwell, Emmanuel Pinteaux

**Affiliations:** 1 Faculty of Life Sciences, A.V. Hill Building, University of Manchester, Oxford Road, Manchester, M13 9PT, UK; 2 Immunology Research Centre, School of Biochemistry and Immunology, Trinity Biomedical Sciences Institute, Trinity College Dublin, Dublin 2, Ireland; 3 Laboratory of Molecular Neuroendocrinology, Institute of Experimental Medicine, Budapest, H-1450, Hungary

**Keywords:** Neurons, Toll-like receptors, Lipopolysaccharide, Neutrophils, Chemokines, Endothelial cells

## Abstract

**Background:**

The innate immune response in the brain is initiated by pathogen-associated molecular patterns (PAMPS) or danger-associated molecular patterns (DAMPS) produced in response to central nervous system (CNS) infection or injury. These molecules activate members of the Toll-like receptor (TLR) family, of which TLR4 is the receptor for bacterial lipopolysaccharide (LPS). Although neurons have been reported to express TLR4, the function of TLR4 activation in neurons remains unknown.

**Methods:**

TLR4 mRNA expression in primary mouse glial and neuronal cultures was assessed by RT-PCR. Mouse mixed glial, neuronal or endothelial cell cultures were treated with LPS in the absence or the presence of a TLR4 specific antagonist (VIPER) or a specific JNK inhibitor (SP600125). Expression of inflammatory mediators was assayed by cytometric bead array (CBA) and ELISA. Activation of extracellular-signal regulated kinase 1/2 (ERK1/2), p38, c-Jun-N-terminal kinase (JNK) and c-Jun was assessed by Western blot. The effect of conditioned media of untreated- versus LPS-treated glial or neuronal cultures on endothelial activation was assessed by neutrophil transmigration assay, and immunocytochemistry and ELISA were used to measure expression of intercellular cell adhesion molecule (ICAM-1) and vascular cell adhesion molecule (VCAM-1).

**Results:**

LPS induces strong release of the chemokines RANTES and CXCL1 (KC), tumor necrosis factor-α (TNFα) and IL-6 in primary mouse neuronal cultures. In contrast, LPS induced release of IL-1α, IL-1β and granulocyte-colony stimulating factor (G-CSF) in mixed glial, but not in neuronal cultures. LPS-induced neuronal KC expression and release were completely blocked by VIPER. In glial cultures, LPS induced activation of ERK1/2, p38 and JNK. In contrast, in neuronal cultures, LPS activated JNK but not ERK1/2 or p38, and the specific JNK inhibitor SP600125 significantly blocked LPS-induced KC expression and release. Finally, conditioned medium of LPS-treated neuronal cultures induced strong expression of ICAM-1 and VCAM-1 on endothelial cells, and induced infiltration of neutrophils across the endothelial monolayer, which was inhibited by VIPER.

**Conclusion:**

These data demonstrate for the first time that neurons can play a role as key sensors of infection to initiate CNS inflammation.

## Background

Tissue infection or injury triggers innate immunity, which is the first line of defense against invading pathogens leading to initiation of inflammation, clearance of pathogen and tissue repair. This response is initiated via recognition of pathogen-associated molecular patterns (PAMPS) by pathogen recognition receptors (PRRs) such as the Toll-like receptors (TLRs) (see [1] for review). This family of receptors has been characterized for their homology with the Toll receptor of *Drosophila* that is involved in dorso-ventral patterning during fly development. The innate immune response is also initiated upon tissue injury in the absence of infection, a mechanism known as sterile inflammation, during which injured cells release endogenous molecule messengers, called danger-associated molecular patterns (DAMPS) that can also activate TLRs [2,3]. To date, 13 TLR isoforms have been identified in mammals, each of which recognizes specific PAMPS or DAMPS [4]. Specifically, TLR4 is the receptor for bacterial lipopolysaccharide (LPS), and its activation by LPS leads to the activation of mitogen-activated protein kinases (MAPKs), extracellular-signal regulated kinase 1/2 (ERK1/2), p38 and c-Jun-N-terminal kinase (JNK) in circulating and tissue-specific macrophages [5]. Activation of TLRs leads to macrophage activation, characterized by expression of major histocompatibility complex (MHC) class I and II molecules, expression of various cytokines and chemokines and initiation of adaptive immune response and inflammation [6].

The innate immune response in the brain occurs in response to central nervous system (CNS) infection or injury, such as meningitis, stroke or brain trauma (see [7] for review). It is characterized by a rapid activation of microglia (brain-specific macrophages), activation of the brain endothelium, expression of various pro-inflammatory mediators (including cytokines and chemokines), and subsequent neutrophil infiltration into the brain tissue [8]. TLR4 activation mediates central inflammation in response to CNS infection and injury, and we found recently that ischemic brain damage is significantly reduced in *TLR4* deficient mice compared to wild type mice [9]. Although the role of TLR4 activation in microglia has been investigated extensively, there has been debate regarding TLR4 expression by other brain cells. Early studies found that TLR4 is expressed primarily by microglia but not by astrocytes or neurons [10,11]. In contrast, other studies found that brain cells, and in particular neurons, can express TLR4 [12-14], and TLR signaling (including TLR4) in neuronal cells regulates neural precursor cell proliferation, axonal growth, neuronal plasticity and adult neurogenesis (see [15] for review). However, the role of neuronal TLR4 in CNS innate immunity remains completely unknown.

Here we demonstrate for the first time that neuronal TLR4 activation by LPS *in vitro* induces strong expression of neuronal chemokines in a JNK-dependent manner, and triggers endothelial activation and subsequent neutrophil trans-endothelial migration. These data demonstrate for the first time that neuronal TLR4 activation can play a key role in the initiation of innate immunity during CNS infection or injury.

## Methods

### Animals and reagents

This study used C57BL/6 mice that were housed at 21°C ± 1°C, 55% ± 10% humidity and maintained in a 12 hour light–dark cycle with free access to food and water. All animals were used according to the Animals (Scientific Procedures) Act (UK) 1986, and were euthanized according with our Project Licence (PL40/3076) approved by the Home Office (UK). Cell culture reagents were purchased from Invitrogen (Paisley, UK), Sigma (Gillingham, UK), and Biowhittaker (Wokingham, UK). Fetal bovine serum (FBS) was obtained from PAA Laboratories (Dartmouth, UK) and plasma-derived serum (PDS) was from First Link Ltd (Wolverhampton, UK). Ultrapure LPS (*Escherichia coli* 0111:B4) was purchased from Invitrogen. The specific TLR4 antagonist VIPER and its control peptide (CP7) were provided by Dr Andrew Bowie (Trinity College Dublin, Ireland). All other reagents were purchased from Sigma unless stated otherwise.

### Primary glial, neuronal and endothelial cell cultures

Primary mixed glial cultures were prepared from the brains of one- to three-day-old mice as described previously [16] using (D)MEM supplemented with 10% FBS, 1 U/ml penicillin and 1 μg/ml streptomycin, and grown in a humidified incubator at 37°C with 5% CO_2_, 95% air until reaching confluency (14 to 20 days *in vitro* (DIV)). Astrocytes or microglia were extracted and purified from mixed glial cultures, and cultured in (D)MEM supplemented with 10% FBS, 1 U/ml penicillin and 1 μg/ml streptomycin as previously reported [16].

Primary neuronal cell cultures were prepared from the brains of mouse embryos at 14 to 16 days of gestation as described previously [17]. Cells were seeded at a density of 6 x 10^5^ cells/ml onto poly-D-lysine (PDL)-coated tissue culture plates in neurobasal medium containing 1 U/ml penicillin, 1 μg/ml streptomycin, 1% glutamine, 5% PDS, 2% B27 without antioxidants and 20 μM 5’-fluoro-2-deoxyuridine (FUDR) to inhibit glial cell growth. Cultures were grown in a humidified incubator at 37°C with 5% CO_2_, 95% air until 12 DIV, and were composed of 99% neurons with less than 1% glial contamination, as assessed by immunocytochemistry (not shown).

Primary cultures of mouse brain endothelial cells were prepared from the brains of 4- to 12- week-old C57BL/6 mice as previously reported [18]. Endothelial cells were grown in medium consisting of (D)MEM-F12, 10% PDS, 10% FBS, 100 μg/ml of endothelial cell growth supplement (BD Biosciences, Oxford, UK), 100 μg/ml heparin, 2 mM glutamine, 1 U/ml penicillin and 1 μg/ml streptomycin, and were used when cultures reached confluency (14 DIV). The purity of endothelial cultures was close to 100%, as characterized by Zona Occludens-1 and Von-Willebrand factor immunocytochemistry (data not shown).

### Reverse transcriptase polymerase chain reaction

Total RNA was extracted using Trizol® Reagent (Invitrogen) according the manufacturer’s instructions, and 1 μg of total RNA was then reverse transcribed with Moloney murine leukemia virus reverse transcriptase (Invitrogen) for 1 hour at 37°C. PCR amplification of 2 μl of cDNA was performed using a ReadyMixTM Taq PCR Reaction Kit (Sigma) with 10 pM of specific forward and reverse primers for TLR4 [19] and glyceraldehyde 3-phosphate dehydrogenase (GAPDH) as housekeeping gene (primers sequences and amplification programs are available upon request). The amplified cDNAs (481 bp for TLR4 and 239 bp for GAPDH) were visualized on a 1.5% agarose gel by electrophoresis at 100 V for 60 min, and the image was captured using an Image Quant 350 camera (GE healthcare, Cardiff, UK).

### Cell treatments and sample preparation

Cultures were treated with LPS (0.1 to 100 ng/ml diluted in PBS or dimethyl sulfoxide (DMSO)) for 15 to 120 min (for ERK1/2, p38, JNK and c-Jun activation) or 24 hours (for inflammatory mediator expression and neutrophil transmigration experiments). To study the involvement of neuronal TLR4 signaling on the expression of the chemokine CXCL1 (KC) and neutrophil transmigration, neurons were pre-incubated with TLR4 specific antagonist (VIPER) or control peptide (CP7) diluted in PBS, 30 min prior to treatment with LPS. The involvement of the JNK signaling pathway in LPS actions in neurons was assessed by treating cultures with DMSO alone or with a specific JNK inhibitor (SP600125, diluted in DMSO), 30 min prior to treatment with LPS (diluted in DMSO).

### Inflammatory mediator expression

Expression levels of inflammatory mediators including TNFα, regulated upon activation normal T-cell expressed and presumably secreted (RANTES, CCL5), KC, IL-6, IL-1α, IL-1β and granulocyte colony-stimulating factor (G-CSF), were assayed using a mouse-specific cytometric bead array (CBA) (BD Biosciences, UK) according to the manufacturer’s instructions.

KC, intercellular cell adhesion molecule (ICAM-1) and vascular cell adhesion molecule (VCAM-1) expression levels were assayed using an ELISA kit (R&D Systems, Abingdon, UK). Standards and samples (100 μl) were assayed in duplicate. The absorbance was measured by using a plate reader (MRX, Dynatech, Willenhall, UK) and results were calculated from the standard curve. The minimum detection limit was 13 pg/ml for KC ELISA and 16 pg/ml for ICAM-1 and VCAM-1 ELISAs.

### Western blot analysis

Activation of ERK1/2, JNK, p38 and c-Jun was assessed by Western blot analysis using total and phosphorylated specific antibodies (New England Biolabs, Hitchin, UK) diluted 1:1000 in Tween-PBS containing 1% BSA, followed by incubation with a secondary horseradish peroxidase (HRP)-conjugated anti-rabbit antibody (DAKO, Glostrup, Denmark) diluted 1:500 in 10% non fat dry milk in Tween-PBS, as previously described [16]. Detection of the secondary antibody was done by exposing the membrane to an Image Quant 350 camera. Images (for ERK1/2, p38 and JNK) were analyzed semi-quantitatively by Image Quant TL 7.0 image analysis software (GE healthcare, UK), and values were expressed as fold increase compared to basal MAPK activity detected in untreated cultures.

### Bone marrow-derived neutrophil isolation

Freshly isolated neutrophils were obtained from male C57BL/6 mice euthanized by CO_2_ inhalation. Bone marrows were flushed from femurs and tibias with 1 to 2 ml of buffer A (1 mM ethylenediaminetetraacetic acid (EDTA), 0.1% BSA in PBS) using a 25 G needle. Tissues were homogenized through a 19 G needle and centrifuged at 400 *g* for 10 min. Cells were resuspended in 3 ml of 0.2% NaCl for 30 to 45 sec in order to lyse red blood cells, and osmolarity was restored by the addition of 7 ml 1.2% NaCl. The suspension was passed through a 30 μm cell strainer, and cells were resuspended in buffer A. Cells were then incubated with anti-Ly6G-biotin antibody and anti-biotin microbeads (Miltenyi Biotech, Bisley, UK) for 10 min at 4°C, and neutrophils were immuno-magnetically separated by passing the cell suspension through an LS column and magnet (Miltenyi). The column was removed from the magnet and the cells were eluted in buffer A.

### Neutrophil trans-endothelial migration assay

Endothelial cells were cultured and grown to confluency on Transwell inserts until DIV14, as stated above. Neutrophils (2 x 10^5^ cells) were added to the luminal (top) compartment of Transwells. Endothelial cultures were then left untreated or were treated with LPS (10 ng/ml, diluted in fresh neuronal or glial medium) or with conditioned medium from LPS (10 ng/ml)-treated neurons or glia (collected directly 24 hours after LPS treatment without washing), in the absence or the presence of VIPER (2 μM) or CP7 (2 μM), added to the abluminal (bottom) compartment of Transwells. After 24 hours, the abluminal (transmigrated) fraction of neutrophils was collected and centrifuged at 800 *g* for 10 min, and neutrophil number was counted using a hemocytometer. Neutrophil transmigration was expressed as a fold increase compared to migration observed under control conditions.

### Immunocytochemistry

Expression of ICAM-1 and VCAM-1 in endothelial cultures was visualized by immunocytochemistry using specific anti-mouse ICAM-1 or VCAM-1 primary antibodies (R&D Systems), followed by Alexa fluor 594-conjugated donkey-anti goat antibody (Invitrogen). Cultures were then washed extensively in PBS and mounted on microscope slides using 4',6-diamidino-2-phenylindole (DAPI)-containing Vectashield mounting medium. Images were acquired using a wide-field fluorescent microscope (Leica) and processed using the Image J software.

### Statistical analysis

Data were collected from a set of 3 to 5 independent experiments, analyzed with GraphPad Prism 5.0, and expressed as mean ± SD. Comparisons between groups of treatments were carried out using one-way analysis of variance (ANOVA) followed by Tukey’s multiple comparison post-hoc test. Data were considered statistically significant when *P* <0.05.

## Results

### TLR4 mRNA is expressed in glial and neuronal cells

**Figure 1:**
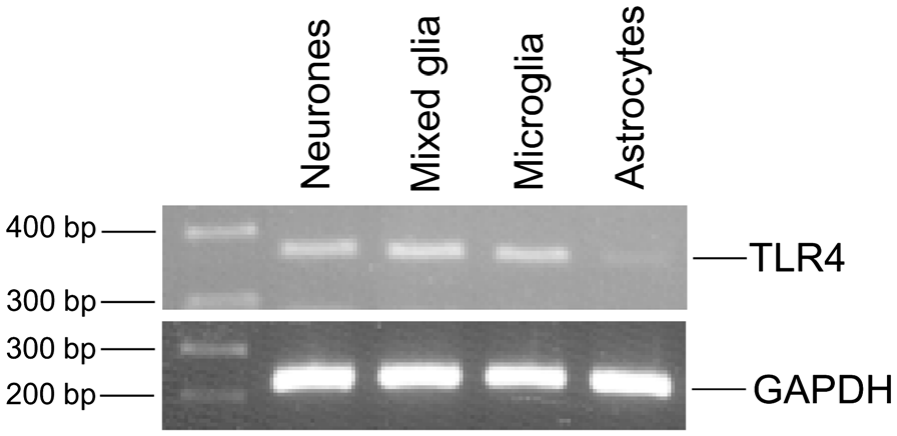
** Expression of TLR4 mRNA in neuronal and glial cell cultures.** Neuronal, mixed glial, microglial and astrocytic primary cultures were analyzed by RT-PCR for expression of TLR4 and GAPDH mRNAs. cDNA amplification was visualized by agarose gel electrophoresis. Images shown are representative of three experiments carried out on separate cultures. GAPDH, glyceraldehyde 3-phosphate dehydrogenase; TLR4, Toll-like receptor 4.

TLR4 mRNA was expressed in mixed glial, microglial and (albeit at much lower levels), in astrocytic cultures (Figure 1), which confirmed previous studies showing that TLR4 expression is predominant in microglial cells [10,11]. TLR4 mRNA expression was also detected in neuronal cultures (at levels similar to those of microglia), which confirmed published studies demonstrating that TLR4 is also constitutively expressed in neuronal cells [15]. 

### LPS induces expression and release of chemokines RANTES and KC, and cytokines TNFÎ± and IL-6 in neuronal cultures

**Figure 2:**
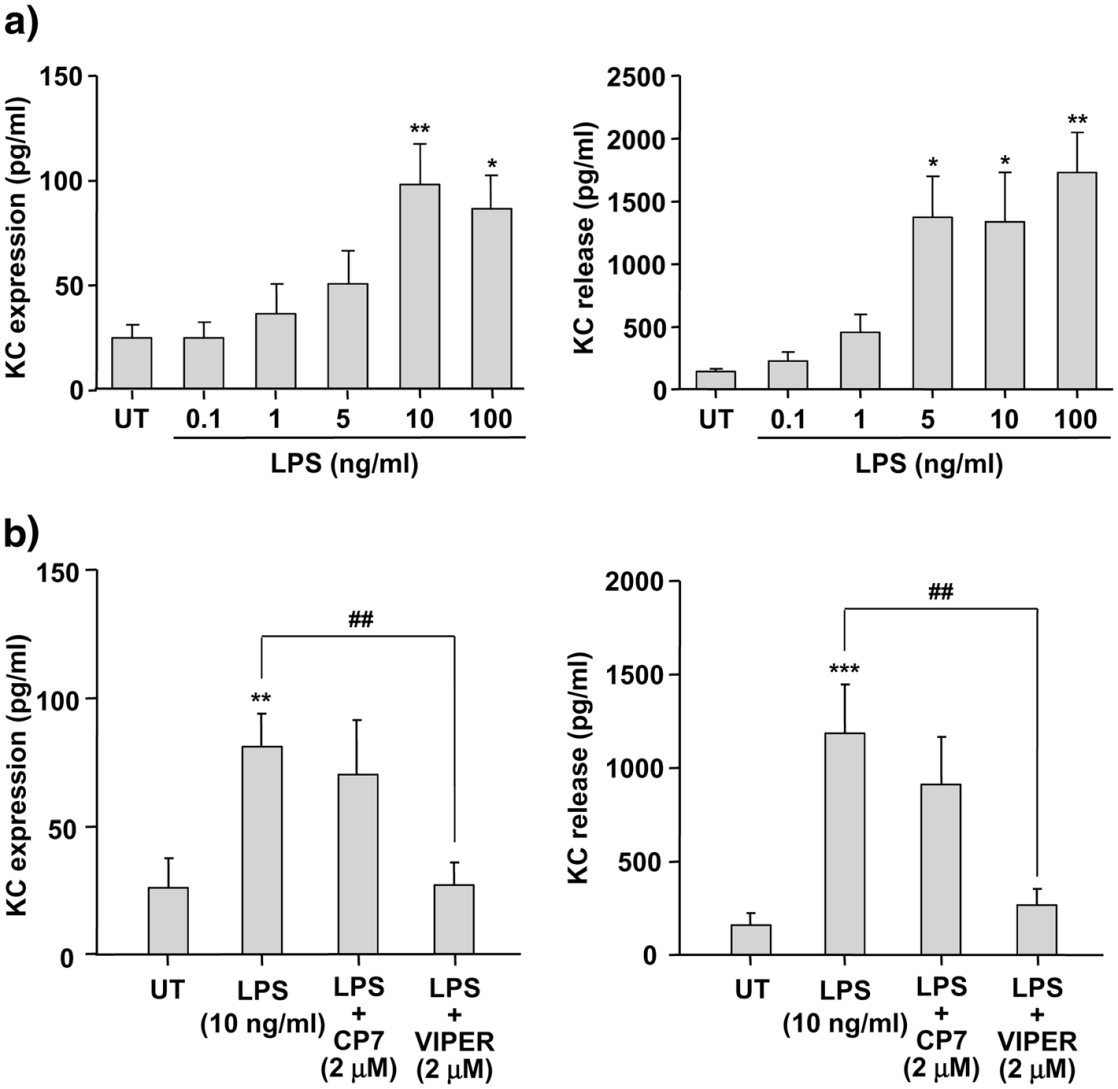
** Effect of LPS on KC expression and release in neuronal cultures.** Neuronal primary cultures were left untreated (UT) or were treated with LPS (0.1 to 100 ng/ml) for 24 hours (**a**). Neuronal cultures were left untreated (UT) or were treated with LPS (10 ng/ml) for 24 hours, in the absence or the presence of the TLR4 antagonist VIPER or control peptide CP7 (2 μM) (**b**). Cell lysates and culture supernatants were assayed for KC levels by ELISA. Data are presented as mean ± SD of at least three independent experiments carried out on separate cultures. *P <0.05, **P <0.01, ***P <0.001, LPS-treated versus untreated cultures, ##*P* <0.01 LPS + VIPER versus. LPS alone, using one-way ANOVA and Tukey’s multiple comparison post-hoc test. ANOVA, analysis of variance; KC, CXCL1; LPS, lipopolysaccharide; TLR4, Toll-like receptor 4.

The role of neuronal TLR4 and LPS actions in neuronal cells remains unknown. We first carried out a CBA analysis on the conditioned media from LPS-treated glial or neuronal cultures to assess expression levels of key inflammatory mediators. In mixed glial cultures, LPS induced strong release of chemokines RANTES (63 ± 5 fold) and KC (135 ± 18 fold), cytokines TNFα (529 ± 43 fold), IL-6 (2097 ± 61 fold), IL-1α (5 ± 1 fold), IL-1β (16 ± 8 fold) and G-CSF (437 ± 85 fold) (Table 1). In neuronal cultures, LPS failed to induce IL-1α or IL-1β release, and induced a much lower release of TNFα (4 ± 1 fold), IL-6 (10 ± 2 fold) and G-CSF (2 ± 0.1 fold) than in glial cultures. Importantly, LPS-induced RANTES release from neurons was high (20 ± 2 fold), and the increase in KC release in response to LPS was much greater in neuronal cultures (220 ± 36 fold) than in glial cultures (135 ± 18 fold) (Table 1). LPS-induced KC expression and release in neurons occurred in a concentration-dependent manner, with maximum KC expression obtained in response to 10 ng/ml LPS, while maximum KC release was obtained in response to 5, 10 and 100 ng/ml LPS (Figure 2a). Therefore, 10 ng/ml LPS was the concentration chosen for all subsequent experiments.

**Table 1:** Effect of LPS on cytokines and chemokines expression in mixed glial and neuronal cultures

**A) Mixed glial cultures**							
	**TNFα**	**RANTES**	**KC**	**IL-6**	**IL-1α**	**IL-1β**	**G-CSF**
Untreated	11 ± 9	122 ± 44	29 ± 10	14 ± 4	7 ± 3	6 ± 5	10 ± 12
LPS-treated	5,673 ± 468^***^	7,805 ± 679^***^	3,974 ± 547^***^	28,945 ± 837^***^	36.7 ± 9.4^***^	98.5 ± 50.7^***^	4,283 ± 832^***^
**B) Neuronal cultures**							
	**TNFα**	**RANTES**	**KC**	**IL-6**	**IL-1α**	**IL-1β**	**G-CSF**
Untreated	19 ± 16	20 ± 4	2 ± 4	7 ± 2	13 ± 3	13 ± 4	21 ± 6
LPS-treated	85 ± 12^**^	414 ± 42^***^	549 ± 89^***^	70 ± 12^***^	13 ± 4	14 ± 3	38 ± 3^**^

Mixed glial (**A**) and neuronal (**B**) primary cultures were treated with LPS (100 ng/ml) for 24 hours, and culture supernatants were assayed for various cytokines and chemokines by CBA analysis. Data are presented as mean ± SD of three independent experiments carried out on separate cultures. **P <0.01, ***P <0.001, LPS-treated versus untreated cultures, using one-way ANOVA and Tukey’s multiple comparison post-hoc test. ANOVA, analysis of variance; CBA, cytometric bead array; G-CSF, colony stimulating factor; KC, CXCL1; LPS, lipopolysaccharide; RANTES, regulated upon activation normal T-cell expressed and presumably secreted; TNFα, tumor necrosis factor-α.

In order to confirm that LPS-induced KC expression and release was mediated by the action of LPS on its receptor (namely TLR4), we tested the effect of a specific TLR4 antagonist VIPER and its control peptide (CP7) on KC expression and release in neuronal cultures. VIPER added at 2 μM (optimum concentration determined in concentration-dependent experiments, not shown), 30 min prior to LPS treatment, completely abolished LPS-induced KC expression and release (Figure 2b), while VIPER alone had no effect (not shown). In contrast, the control peptide CP7 (2 μM) added prior to LPS treatment had no effect on LPS-induced KC expression and release (Figure 2b). VIPER or CP7 (added at 2 μM) had no effect on neuronal cell death or viability, as assessed by lactate dehydrogenase (LDH) release or methylthiazol-tetrazolium (MMT) assay, respectively (not shown).

### LPS-induced KC expression and release in neuronal cultures is dependent on activation of the JNK pathway, but is independent of ERK1/2 or p38

**Figure 3:**
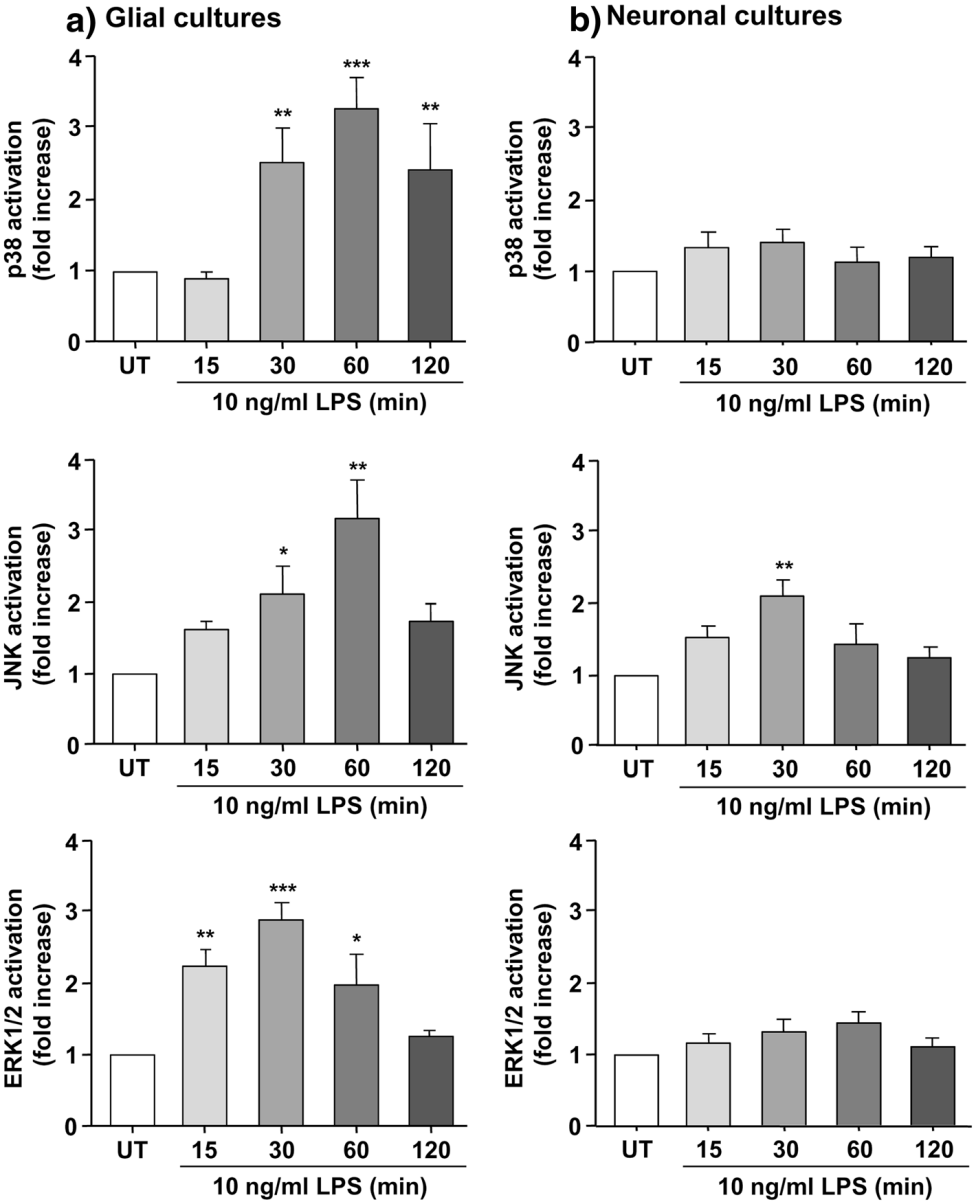
** Effect of LPS on activation of p38, ERK1/2 and JNK MAPKs in neuronal and mixed glial cultures.** Glial (**a**) or neuronal (**b**) primary cultures were left untreated (UT) or were treated with LPS (10 ng/ml) for 15, 30, 60 or 120 min. Cell lysates were assayed for p38, ERK1/2 and JNK activation by Western blot analysis. Levels of phosphorylated MAPKs were analyzed semi-quantitatively, normalized relative to total MAPKs, and presented as fold increase compared to untreated cultures. Data are presented as mean ± SD of at least three independent experiments carried out on separate cultures. *P <0.05, **P <0.01, ***P <0.001, LPS-treated versus untreated cultures, using one-way ANOVA and Tukey’s multiple comparison post-hoc test. ANOVA, analysis of variance; ERK1/2, extracellular-signal regulated kinase 1/2; JNK, c-Jun-N-terminal kinase; LPS, lipopolysaccharide; MAPK, mitogen-activated protein kinase.

To investigate the signaling pathways involved in the mechanism of neuronal KC expression induced by LPS, we assessed activation of the MAPKs, ERK1/2, JNK and p83 in glial and neuronal cultures. In glial cultures, LPS induced strong activation of ERK1/2, JNK and p38 in a time-dependent manner, with maximum activation of p38 and JNK detected at 60 min, and ERK1/2 detected at 30 min ( 3a). While p38 activation remained significantly elevated 120 min after treatment with LPS, JNK and ERK1/2 activity returned to basal levels at 120 min. In neuronal cultures, LPS failed to induce activation of p38 or ERK1/2, but induced significant activation of JNK, 30 min after LPS treatment (level of activation detected was similar to that detected in glial cultures) (Figure 3b). JNK activity returned to basal levels 60 min after LPS treatment, as opposed to glial cultures where JNK activity remained high up to 120 min.

**Figure 4:**
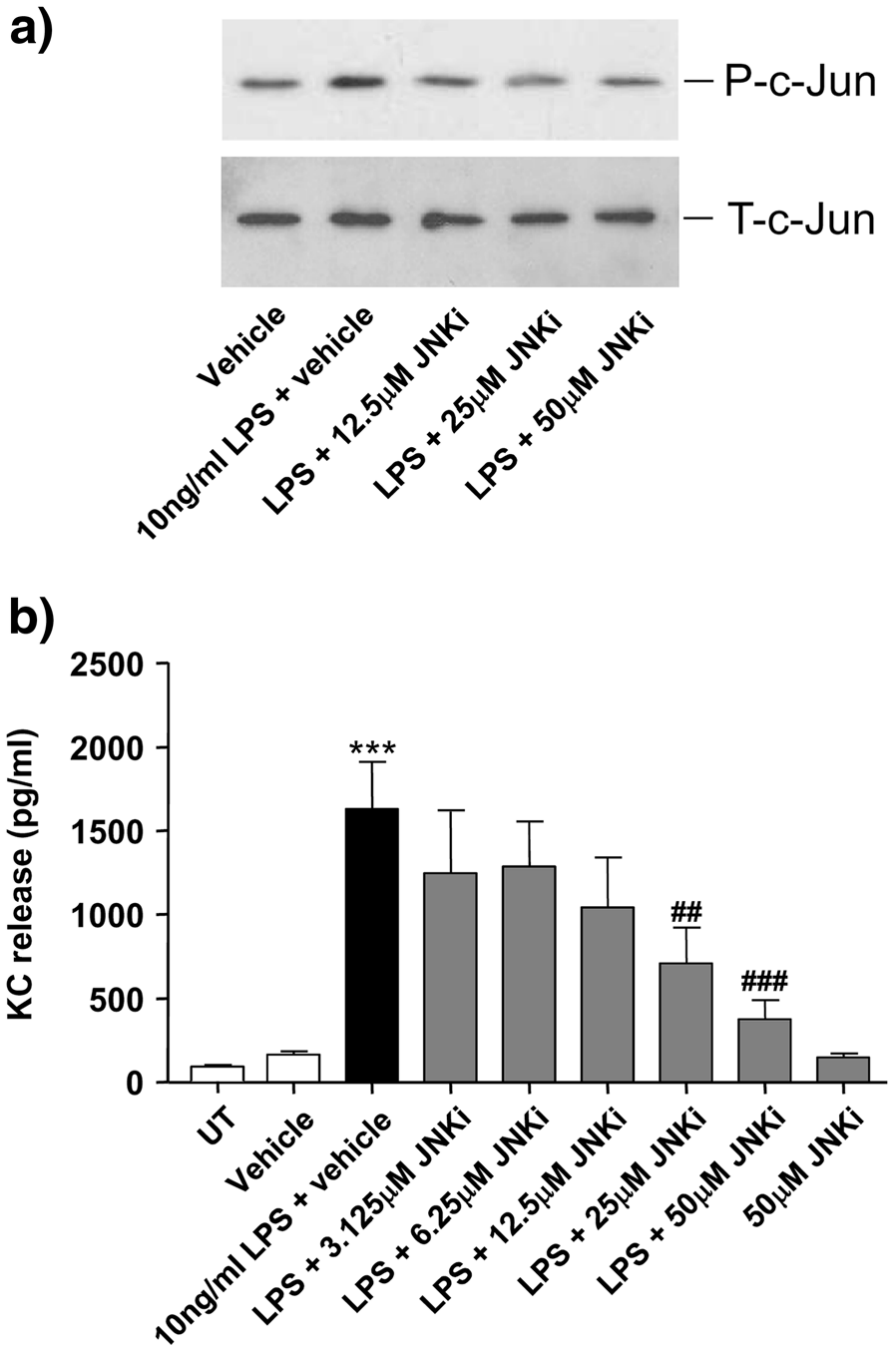
** Effect of JNK inhibition on c-Jun activity and LPS-induced KC release in neuronal cultures.** Neuronal primary cultures were left untreated (UT), were treated with vehicle (DMSO), or were treated with LPS (10 ng/ml prepared in DMSO) for 30 min (for c-Jun activity, levels of phosphorylated (P-c-Jun) compared to total (T-c-Jun) c-Jun, **a**) or for 24 hours (for KC release, **b**) in the absence or the presence of increasing concentrations of a specific JNK inhibitor (SP600125, JNKi) added 30 min prior to treatment with LPS. Cell lysates were assayed for c-Jun activity by Western blot analysis. Image shown is representative of three experiments carried out on separate cultures (**a**). Culture supernatants were assayed for KC levels by ELISA (**b**). Data are presented as mean ± SD of at least three independent experiments carried out on separate cultures. **P <0.01, ***P <0.001, LPS + JNKi-treated versus LPS-treated cultures, using one-way ANOVA and Tukey’s multiple comparison post-hoc test. ANOVA, analysis of variance; DMSO, dimethyl sulfoxide; JNK, c-Jun-N-terminal kinase; KC, CXCL1; LPS, lipopolysaccharide.

These observations suggest that LPS induces specific activation of the JNK signaling pathway in neuronal cells. To determine whether JNK mediates KC synthesis in neuronal cells, we tested the effect of a specific JNK inhibitor (SP600125) on LPS-induced KC release in neuronal cultures. JNK inhibition with SP600125 blocked LPS-induced c-Jun activity, which is a direct and specific downstream target of JNK activity (Figure 4a). As mentioned above, LPS induced significant release of KC from neuronal cultures after 24 hours of treatment (Figure 4b). The specific JNK inhibitor SP600125, added 30 min prior to LPS treatment, strongly inhibited LPS-induced KC release in a concentration-dependent manner, with significant inhibition detected at 25 and 50 μM SP600125 (Figure 4b). DMSO or SP600125 alone had no effect on KC release (Figure 4b), and no effect of DMSO or SP600125 on neuronal viability was detected (not shown).

### Conditioned medium of LPS-treated neuronal cultures induces endothelial cell activation and neutrophil trans-endothelial migration

**Figure 5:**
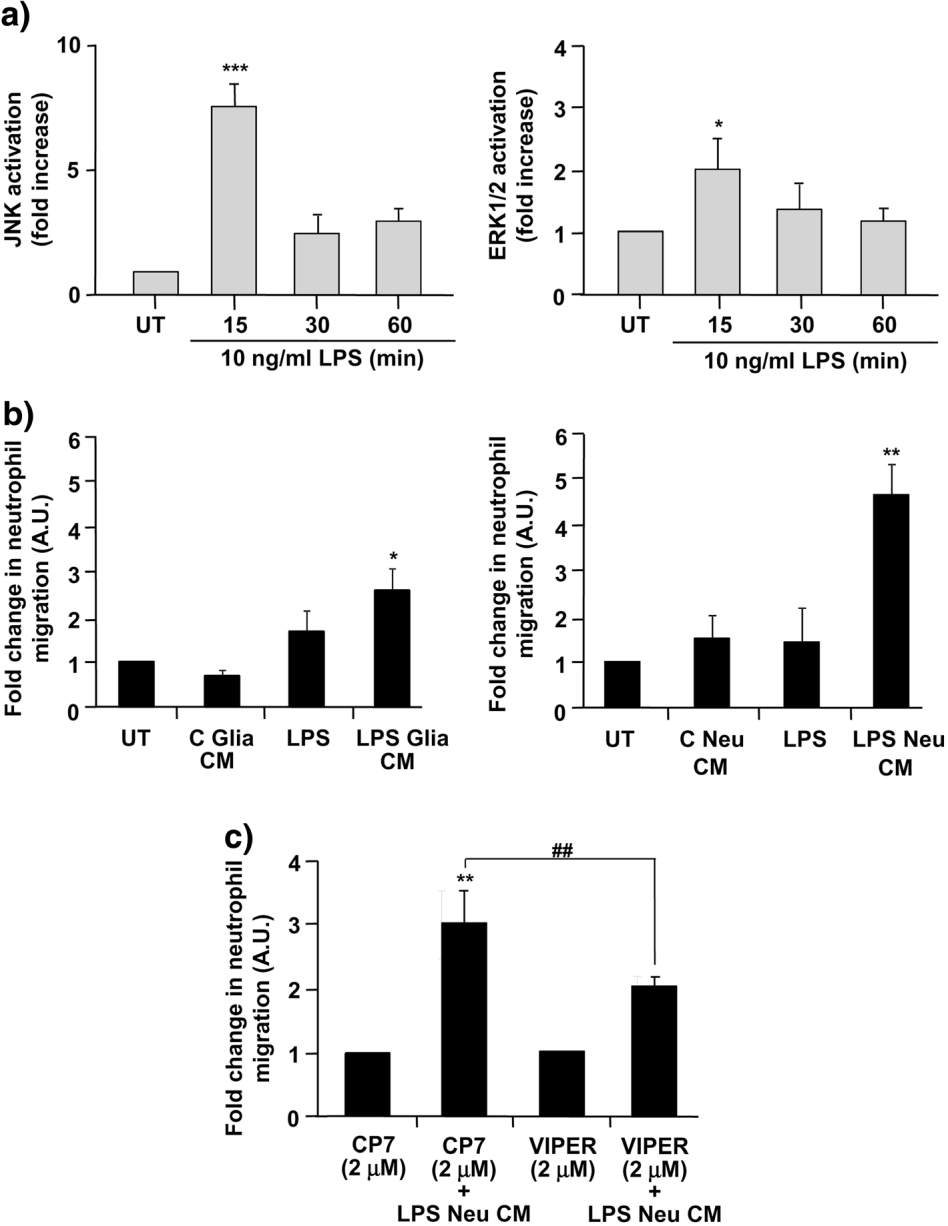
** Effect of LPS and conditioned medium of LPS-treated glial or neuronal cultures on MAPKs activation in endothelial cells and neutrophil transmigration.** Endothelial primary cultures were left untreated (UT) or were treated with LPS (10 ng/ml) for 15, 30 or 60 min. Cell lysates were assayed for JNK and ERK1/2 activation by Western blot analysis. Levels of phosphorylated MAPKs were analyzed semi-quantitatively, normalized relative to total MAPKs, and represented as fold increase compared to untreated cultures (**a**). Endothelial primary cultures were left untreated (UT), were treated with LPS, or were treated with conditioned medium of untreated glial (C Glia CM) or neuronal cultures (C Neu CM), or conditioned medium of LPS (10 ng/ml for 24 hours)-treated glial (LPS Glia CM) or LPS (10 ng/ml for 24 hours)-treated neurons (LPS Neu CM), and neutrophil trans-endothelial migration was assayed (**b**). Endothelial primary cultures were treated with conditioned medium of neuronal cultures treated with VIPER or CP7 (2 μM) in the absence or the presence of LPS (10 ng/ml) (LPS Neu CM), and neutrophil trans-endothelial migration was assayed (**c**). Data are presented as mean ± SD of three independent experiments carried out on separate cultures. *P <0.05, **P <0.01, ***P <0.001 LPS-treated versus untreated cultures, ##*P* <0.01 LPS + VIPER versus LPS + CP7, using one-way ANOVA and Tukey’s multiple comparison post-hoc test. ANOVA, analysis of variance; ERK1/2, extracellular-signal regulated kinase1/2; JNK, c-Jun-N-terminal kinase; LPS, lipopolysaccharide; MAPK, mitogen-activated protein kinase.

Brain endothelium activation and neutrophil extravasation into the brain tissues are classical hallmarks of brain inflammation in response to CNS injury and/or infection. We, therefore, investigated whether LPS actions on neurons influence brain endothelial cells and subsequent neutrophil transmigration. LPS added directly to endothelial cultures induced significant activation of JNK and ERK1/2 (Figure 5a). This increase in JNK and ERK1/2 activity was maximal 15 min after LPS treatment, while activity of both MAPKs decreased at 30 and 60 min to reach basal levels. These actions of LPS on endothelial cells failed to induce neutrophil transmigration across the endothelium monolayer (Figure 5b). Basal level of neutrophil migration across the control (untreated) endothelial monolayer was low (1.7%). In contrast, conditioned medium (CM) of LPS-treated glial cultures (LPS Glia CM) induced a 2.7-fold increase in neutrophil transmigration, while conditioned medium of LPS-treated neuronal cultures (LPS Neu CM) induced neutrophil transmigration to a much higher level (4.7-fold) (Figure 5b). Conditioned medium of untreated glial (C Glia CM) or neuronal (C Neu CM) cultures had no effect. The effect of conditioned medium of LPS-treated neuronal cultures was significantly reduced in the presence of VIPER (added on neuronal cultures prior to LPS treatment), compared to conditioned medium of LPS-treated neuronal cultures in the presence of the control peptide CP7 (Figure 5c). CP7 or VIPER alone had no effect on neutrophil trans-endothelial migration.

**Figure 6:**
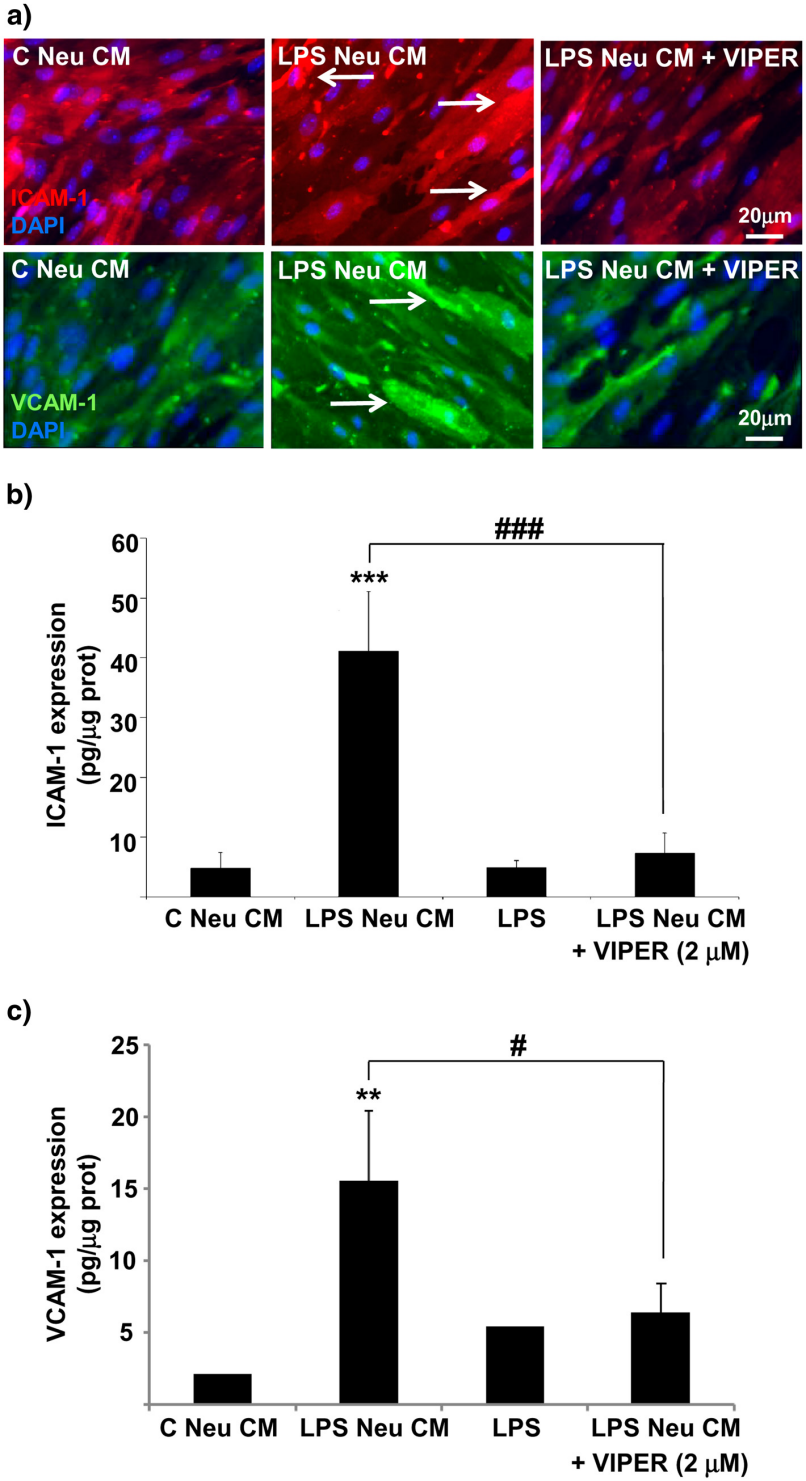
** Effect of neuronal LPS signaling on endothelial adhesion molecules expression.** Endothelial primary cultures were treated with LPS (10 ng/ml), conditioned medium of untreated (C Neu CM) or LPS-treated (LPS Neu CM) neuronal cultures, in the absence or the presence of VIPER (2 μM). Cells were imaged for ICAM-1 and VCAM-1 expression by immunocytochemistry (**a**). Arrows show increased cellular expression of ICAM-1 and VCAM-1. Cell lysates were assayed for ICAM-1 expression and VCAM-1 expression (**b**) by ELISA. Data are presented as mean ± SD of three independent experiments carried out on separate cultures. **P <0.01, ***P <0.001, LPS-treated versus untreated cultures, #*P* <0.05, ###*P* <0.001, LPS + VIPER versus LPS alone, using one-way ANOVA and Tukey’s multiple comparison post-hoc test. ANOVA, analysis of variance; ICAM-1, intercellular cell adhesion molecule; LPS, lipopolysaccharide; VCAM-1, vascular cell adhesion molecule.

Conditioned medium of LPS-treated neuronal cultures caused increased expression of the cell adhesion molecules ICAM-1 and VCAM-1, compared to conditioned medium of untreated neuronal cultures (Figure 6)a and b. This effect was blocked by VIPER, while VIPER alone had no effect (not shown). Finally, LPS added directly to endothelial cultures had no effect on ICAM-1 and VCAM-1 expression (Figure 6b).

## Discussion

The innate immune response is initiated by TLR family members that are activated by PAMPS or DAMPS in response to infection or injury. This response in the brain is considered to be mediated by non-neuronal cells, primarily by microglial cells, since these cells are the main immune and antigen-presenting cells in the CNS [6]. These cells express high levels of TLR4 and they respond rapidly to LPS *in vitro* and *in vivo* to produce a large array of inflammatory mediators [20]. Our study is the first to demonstrate that neuronal cells can be key sensors of infection since these cells respond to LPS to produce pro-inflammatory chemokines, leading to endothelial cell activation and neutrophil trans-endothelial migration.

Despite early studies demonstrating that neurons do not express TLR4 (see [20] for review), we found that primary cortical neurons in our conditions express TLR4 mRNA, which is in agreement with more recent studies showing that TLR4 is expressed in the neuronal cell lineage. Indeed, constitutive expression of TLR4 has been detected in hippocampal neurons [21], sensory neurons [13], neural stem cells [12] and photoreceptor cells [14]. These observations taken together with our data suggest that neurons can, therefore, sense bacterial infection (and possibly endogenous DAMPs), and we demonstrated here that neurons synthesize chemokines (that is, RANTES and KC) as well as the cytokines TNFα and IL-6 (albeit at much lower levels) in response to LPS. Furthermore, we found that LPS-induced KC synthesis in neurons is dependent on TLR4 activation since this response was blocked by a specific TLR4 antagonist (VIPER). The inflammatory responses observed in neuronal cultures may be due to residual glial contamination, but TLR4 mRNA expression was high in our neuronal cultures implying that neurons can indeed sense and respond to LPS. Furthermore, increased KC release in response to LPS was much higher in neuronal cultures (220 fold) than in glial cultures (135 fold), while LPS-induced IL-1α, IL-1β and G-CSF expression (which is thought to be a glial specific response) was very low or absent in neuronal cultures, and this correlated with the very low level of glial contamination in our neuronal cultures. LPS-induced p38 and ERK1/2 activation found in glial cultures was not detected in neuronal cultures, while LPS induced strong activation of JNK in glial but also in neuronal cultures. These data strongly suggest that LPS specifically induces neuronal cells to express chemokines in a JNK-dependent manner, and we showed that LPS-induced KC release in neurons was completely abrogated in the presence of a specific JNK inhibitor.

Our data confirm other studies demonstrating that neurons can respond to TLR ligands but are the first to demonstrate that neurons can produce inflammatory mediators including neuronal chemokine KC in a JNK-dependent manner. A recent study found that TLR4 activation by high-mobility group box-1 (HMGB-1, a well-known TLR4 endogenous ligand) is involved is seizures [22], while another study found that neuronal KC is significantly increased during epilepsy in the rat [23]. These data highlight the relevance of neuronal KC triggered by TLR4 signaling in CNS disorders, which could also play a key role during infection that occurs during stroke [24].

We have further demonstrated that LPS signaling in neurons induces soluble mediators that can activate endothelial cells to express key cell adhesion molecules such as ICAM-1 and VCAM-1, which correlated with increased neutrophil trans-endothelial migration. Indeed, conditioned medium from LPS-treated neuronal cultures induced strong ICAM-1 or VCAM-1 expression as well as neutrophil trans-endothelial migration, and these responses were dependent on neuronal TLR4 activation. LPS (added at 10 ng/ml) could directly activate endothelial cells, measured by increased JNK and ERK1/2 activation, although this effect was transient with no effect on endothelial ICAM-1 or VCAM-1 expression. Furthermore, conditioned medium from LPS-treated neuronal cultures induced neutrophil trans-endothelial migration (unlike LPS alone) and this effect was much higher than that seen with conditioned medium of LPS-treated glial cultures. We therefore demonstrate here a key mechanism by which neurons sensing infection or injury could trigger endothelial activation, recruitment of peripheral immune cells to initiate brain innate immunity. The likely advantage of neurons sensing infection over microglial cells sensing infection alone could be the development of a rapid host response via direct activation of the peripheral autonomic and/or neuroendocrine systems, rapid activation of neurons located in the circumventricular organs (CVO), as well as neurovascular coupling regulated by neurons in response to peripheral / central infection. Although LPS was used in our study to induce TLR4 activation, its relevance to brain injury remains controversial. Indeed, other endogenous TLR4 ligands released during injury, such as HMGB1 or heat shock protein 70 (HSP70) [25,26], could induce similar responses to those observed here, although this remains to be determined.

Although we demonstrated that LPS induces expression of the chemokines RANTES and KC in neurones, the nature of neuronal mediators that trigger endothelial activation and neutrophil trans-endothelial migration in our experiments remains to be investigated. Because the role of chemokines in the activation of brain endothelia is very well documented, the role of neuronal KC (but possibly other mediators) in the activation of endothelial cells here is a likely mechanism, although this remains to be confirmed. Our data demonstrate for the first time that along with a microglial response to LPS, neurons can also respond to LPS and be important players in the initiation of the inflammatory response to infection or injury in the brain. These discoveries identify new targets for the therapeutic treatment of inflammatory-mediated CNS disorders.

## Abbreviations

ANOVA: analysis of variance; BSA: bovine serum albumin; CBA: cytometric bead array; CVO: circumventricular organs; CNS: central nervous system; DAMPS: danger-associated molecular patterns; DAPI: 4',6-diamidino-2-phenylindole; DMSO: dimethyl sulfoxide; EDTA: ethylenediaminetetraacetic acid; ERK1/2: extracellular-signal regulated kinase1/2; FBS: fetal bovine serum; FUDR: 5’-fluoro-2-deoxyuridine; GAPDH: glyceraldehyde 3-phosphate dehydrogenase; G-CSF: colony stimulating factor; HMGB1: high-mobility group box-1; HSP70: heat shock protein 70; HRP: horseradish peroxidase; ICAM-1: intercellular cell adhesion molecule; JNK: c-Jun-N-terminal kinase; KC: CXCL1; LDH: lactate dehydrogenase; LPS: lipopolysaccharide; MAPK: mitogen-activated protein kinase; MHC: major histocompatibility complex; MTT: methylthiazol-tetrazolium; PAMPS: pathogen-associated molecular patterns; PDL: poly-D-lysine; PDS: plasma-derived serum; PRR: pathogen recognition receptors; RANTES: regulated upon activation normal T-cell expressed and presumably secreted; TLR4: Toll-like receptor 4; TNFα: tumor necrosis factor-α; VCAM-1: vascular cell adhesion molecule.

## Competing interests

The authors declare that they have no competing interests.

## Authors’ contributions

EP, NJR and AGB designed the study. SLD, CA, AD, ON and SM performed the experiments and analyzed the data. EP, AD and NJR wrote the manuscript. All authors have read and approved the final version of the manuscript.
